# Elevated levels of circulating IL-7 and IL-15 in patients with early stage prostate cancer

**DOI:** 10.1186/1479-5876-9-162

**Published:** 2011-09-26

**Authors:** Chantal Mengus, Clémentine Le Magnen, Emanuele Trella, Kawa Yousef, Lukas Bubendorf, Maurizio Provenzano, Alexander Bachmann, Michael Heberer, Giulio C Spagnoli, Stephen Wyler

**Affiliations:** 1 ICFS, Departments of Surgery and Biomedicine, Basel University Hospital, Basel, Switzerland; 2 Institute of Pathology, Basel University Hospital, Basel, Switzerland; 3 Oncology Research Unit, Division of Urology and Division of Surgical Research, Zurich University Hospital, Zurich, Switzerland; 4 Department of Urology, Basel University Hospital, Basel, Switzerland

## Abstract

**Background:**

Chronic inflammation has been suggested to favour prostate cancer (PCA) development. Interleukins (IL) represent essential inflammation mediators. IL-2, IL-7, IL-15 and IL-21, sharing a common receptor γ chain (c-γ), control T lymphocyte homeostasis and proliferation and play major roles in regulating cancer-immune system interactions. We evaluated local IL-2, IL-7, IL-15 and IL-21 gene expression in prostate tissues from patients with early stage PCA or benign prostatic hyperplasia (BPH). As control, we used IL-6 gene, encoding an IL involved in PCA progression. IL-6, IL-7 and IL-15 titres were also measured in patients' sera.

**Methods:**

Eighty patients with BPH and 79 with early (1 to 2c) stage PCA were enrolled. Gene expression in prostate tissues was analyzed by quantitative real-time PCR (qRT-PCR). Serum IL concentrations and acute phase protein titres were evaluated by ELISA. Mann-Whitney, Wilcoxon and χ^2 ^tests were used to compare IL gene expression and serum titers in the two groups of patients. Receiver operating characteristic (ROC) curves were constructed to evaluate the possibility to distinguish sera from different groups of patients based on IL titers.

**Results:**

IL-2 and IL-21 gene expression was comparably detectable, with low frequency and at low extents, in PCA and BPH tissues. In contrast, IL-6, IL-7 and IL-15 genes were expressed more frequently (p < 0.0001, p = 0.0047 and p = 0.0085, respectively) and to significantly higher extents (p = 0.0051, p = 0.0310 and p = 0.0205, respectively) in early stage PCA than in BPH tissues. Corresponding proteins could be detected to significantly higher amounts in sera from patients with localized PCA, than in those from patients with BPH (p = 0.0153, p = 0.0174 and p = 0.0064, respectively). Analysis of ROC curves indicates that IL-7 (p = 0.0039), but not IL-6 (p = 0.2938) or IL-15 (p = 0.1804) titres were able to distinguish sera from patients with malignancy from those from patients with benign disease. Serum titres of C reactive (CRP), high mobility group B1 (HMGB1) and serum amyloid A (SAA) acute phase proteins were similar in both groups of patients.

**Conclusions:**

Expression IL-7 and IL-15 genes in prostate tissues and corresponding serum titres are significantly increased in patients with early stage PCA as compared with patients with BPH.

## Background

Prostate cancer (PCA) is the second leading cause of cancer-related death in men with a death rate reaching 26.7% for 2001-2005 in United States[1]. Chronic inflammation has been suggested to play a major role in prostate oncogenesis[2]. Furthermore, local and systemic immunosuppression have also been reported in patients bearing PCA[3-6]. The clarification of molecular mechanisms underlying these phenomena might provide novel insights into PCA induction and progression, of potentially high clinical relevance.

Cytokines represent key mediators of inflammation and play pivotal roles in the interaction between immune system and cancer. A number of them have been suggested to be associated with advanced stage PCA[7]. IL-4 and, in particular, IL-6 have been shown to exert antiapoptotic effects on PCA cells, whereas proangiogenic effects of IL-8 have been demonstrated[7].

A group of lymphokines including, among others, IL-2, IL-7, IL-15 and IL-21 share a receptor γ-chain[8]. Common receptor γ-chain (c- γ) cytokines regulate lymphocyte development[9-12] and support CD4+ and CD8+ T cell homeostatic proliferation and functions[13,14]. IL-2, IL-7, IL-15 and IL-21 are of particular interest in cancer immunotherapy[15]. Importantly, IL-2 is FDA approved for the treatment of melanoma and renal cell carcinoma, whereas IL-7 has been used with promising results in phase I/II trials in clinical oncology[16,17]. IL-15 has not yet been used in clinical trials. However, experimental models and "in vitro" evidence suggest that it may represent a cancer treatment with high potential clinical relevance[18-20]. Finally, IL-21 has been shown in murine models to prevent T lymphocyte exhaustion induced by chronic stimulation[21-23]. In phase I/II clinical trials, IL-21 has also shown reproducible antitumor effects[24,25].

Notably, however, elevated IL-7 serum levels have been detected in Hodgkin disease and in ovarian cancers [26-29]. Furthermore, IL-7 has been shown to be produced by breast and colorectal cancer cells[30,31]. IL-15 has also been shown to be produced by colon cancer cells and specific gene expression has been shown to be associated with distant metastases [32]. Moreover, elevated IL-15 serum levels were detected in multiple myeloma[33].

Little is known about IL-2, IL-7, IL-15 and IL-21 gene expression and protein secretion in patients bearing PCA or BPH. Indeed, IL-2 gene expression and IL-15 protein have been detected in BPH tissues[34,35]. Furthermore, IL-7 gene has been found to be expressed in PCA tissues[36,37] and the corresponding protein has been detected in patients' sera. However, no comparative analysis in patients with BPH and PCA has been performed so far and no data are currently available for IL-21 expression in PCA and BPH.

In order to evaluate the potential role of these IL in early phases of prostate oncogenesis, in this study we have evaluated the expression of specific genes in prostatic tissues from patients bearing either localized pT1-2c PCA or BPH and we have measured the titres of circulating proteins in their sera.

Our results indicate that in prostatic tissues from patients bearing early stage PCA, the expression of IL-7 and IL-15 genes is significantly enhanced, as compared to BPH, while no significant differences could be observed in IL-2 or IL-21 gene expression. Accordingly, titres of circulating IL-7 and IL-15 are increased in patients with localized PCA, as compared to patients with BPH. Most interestingly, IL-7 titres might help to discriminate sera from patients with PCA or BPH.

## Patients and methods

### Patients

We investigated a consecutive series of 159 specimens from men diagnosed for BPH or PCA at the Department of Urology of the University Hospital of Basel (Switzerland) from 2007 to 2010. Patients with BPH underwent conventional transurethral resection (TUR-P), while patients with PCA underwent either palliative TUR-P or endoscopic extraperitoneal radical prostatectomy (EERP). Relevant clinical data were collected by reviewing patients' files.

Written informed consent was obtained from patients in accordance with the requirements of the Ethical Committee of Basel (EKBB, Ref.Nr. EK: 176/07).

### Quantification of gene expression in prostatic tissues by quantitative Real-Time PCR

Prostatic tissues were screened for the presence of tumors by an experienced pathologist (L. Bubendorf). Total RNA was extracted by using RNeasy^® ^MiniKit (Qiagen, Basel, Switzerland), deoxyribonuclease I (Invitrogen, Carlsbad, California) treated and reverse transcribed by using M-MLV Reverse Transcriptase (Invitrogen, Carlsbad, California). Quantitative RT-PCR was performed using the TaqMan^® ^Universal PCR Master Mix (Applied Biosystems, Forster City, CA), and the following primers and probes:

GAPDH[38]

Fwd ATGGGGAAGGTGAAGGTCG

Rev TAAAAGCAGCCCTGGTGACC

Probe FAM-CGCCCAATACGACCAAATCCGTTGAC-TAMRA

IL-2[39]

Fwd AACTCACCAGGATGCTCACATTTA

Rev TCCCTGGGTCTTAAGTGAAAGTTT

Probe FAM-TTTTACATGCCCAAGAAGGCCACAGAACT-TAMRA

IL-6[40]

Fwd CAGCCCTGAGAAAGGAGACATG

Rev GGTTCAGGTTGTTTTCTGCCA

Probe FAM-AGTAACATGTGTGAAAGCAGCAAAGAGGCAC-TAMRA

IL-21, IL-7 and IL-15 primers and probes were provided by Assays-on-Demand, Gene Expression Products (Applied Biosystems, Forster City, CA).

Specific gene expression was quantified by using the 2^-ΔΔC^_T _method[41]. Normalization of gene expression was performed using GAPDH as reference gene.

### ELISA assays

Commercial ELISA kits for IL-6, IL-7 and IL-15 were obtained from Pharmingen (San Diego, CA). Detection limit was 1pg/ml for all assays. HMGB1 ELISA kit was obtained from Shino-Test Corporation (Kanagawa, Japan). SAA and CRP ELISA kits were obtained from USB (Swampscott, MA) and Roche Diagnostics Cobas^® ^(Mannheim, Germany), respectively. Detection limits were 1 ng/ml, 0.6 ng/ml, and 0.6 μg/ml, for HMGB1, SAA and CRP, respectively. All assays were performed in accordance to the instructions from the producers.

### Statistical Analysis

Mann-Whitney tests were used to compare mean expression of cytokine genes and cytokine serum titers for independent samples while Wilcoxon test was used for paired samples. When n ≥ 30, Student's T-Tests were used to compare means. Frequencies of specific gene expression in two groups were assessed by χ^2 ^tests. Differences with p < 0.05 were considered significant. Receiver operating characteristic (ROC) curves were created to verify whether cytokine titers could be used to distinguish patients with PCA from patients with BPH [27].

## Results

### Clinico-pathological profiles of the patients

A group including 79 patients (average age ± SEM = 64.3 ± 0.8) undergoing surgical treatment for localized stage 1-2 PCA and a group of 80 patients (average age ± SEM = 68.1 ± 0.9) undergoing surgical treatment for BPH were enrolled in the study. Of the 79 PCA patients, 2 (2.6%) were diagnosed with stage pT1, 2 (2.6%) with stage pT1a, 1 (1.3%) with stage pT1b, 3 (3.8%) with stage pT2, 13 (16.4%) with stage pT2a, 4 (5%) with stage pT2b, 54 (68.3%) with stage pT2c.

Average serum Prostate Specific Antigen (PSA) ± SEM in PCA patients was 8.26 ± 1.46 ng/ml (n = 74) and average Gleason score ± SEM was 6.63 ± 0.10 (n = 78). Average serum PSA value ± SEM in BPH patients was 5.49 ± 0.87 ng/ml (n = 63).

### Expression of c-γ cytokines genes in prostatic tissues

**Figure 1:**
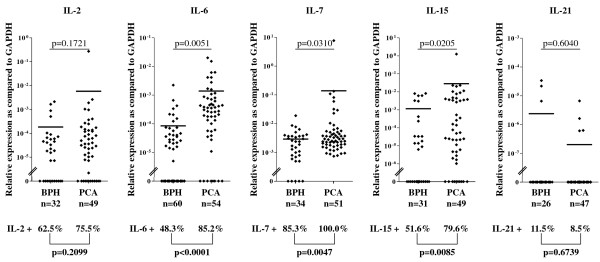
**Expression of IL genes in early stage PCA and BPH tissues**. Total cellular RNA was extracted from BPH and PCA tissues, DNAse treated, reverse transcribed and analyzed by quantitative real-time PCR for IL-2 (BPH: n = 32, PCA: n = 49), IL-7 (BPH: n = 34, PCA: n = 51), IL-15 (BPH: n = 31, PCA: n = 49), IL-6 (BPH: n = 60, PCA: n = 54) and IL-21 (BPH: n = 26, PCA: n = 47) specific gene expression. Data are expressed as ratio to GAPDH gene expression. Mean values (▬) are indicated for each group. Digits in individual panels indicate percentages of samples displaying gene expression levels exceeding detection limits (bold, bottom of the panel) and statistical significance of differential extent of specific gene expression in BPH or PCA specimens (top of the panel).

In the initial phase of our study, we quantitatively analyzed IL-2, IL-7, IL-15 and IL-21 gene expression in a series of tissue samples from localized PCA and BPH. As shown in figure 1, IL-2 gene expression was detectable in 20/32 (62.5%) and in 37/49 (75.5%) BPH and PCA specimens, respectively (p = 0.2099). Under a quantitative view point, comparably low levels of expression could be observed in BPH and PCA tissues (p = 0.1721).

In contrast, IL-7 gene expression was significantly more frequently observed in PCA than in BPH specimens [29/34 (85.3%) and 51/51 (100%) BPH and PCA, respectively (p = 0.0047)]. IL-15 gene was also expressed more frequently in PCA than in BPH samples [16/31 (51.6%) and 39/49 (79.6%) BPH and PCA specimens, respectively (p = 0.0085)]. Notably, under a quantitative point of view, IL-7 and IL-15 gene expression was also significantly increased in tissues from patients bearing early stage PCA as compared to those from patients with BPH (p = 0.0310 and p = 0.0205, respectively, figure 1).

IL-21 gene expression was relatively rarely detectable in prostatic tissues from patients bearing BPH or stage pT1-2c PCA [3/26 (11.5%) and 4/47 (8.5%) BPH and PCA tissues, respectively (p = 0.6739)]. Furthermore, the average level of expression did not significantly differ in the two groups (p = 0.6040, figure 1).

IL-6 is known to be a mediator of PCA morbidity, and may act as a cell growth factor and protect cancer cells from death[42]. Therefore, we used the expression of this gene as control of the integrity of our assays. Indeed, IL-6 gene expression was detected in 29/60 (48.3%) and in 46/54 (85.2%) BPH and early stage PCA specimens, respectively (p < 0.0001). Under a quantitative point of view, IL-6 gene expression was also significantly increased in tissues from patients bearing PCA as compared to BPH (p = 0.0051), thus confirming our previous findings[43].

Taken together these data indicate that although genes encoding the c-γ cytokines under investigation may be expressed in BPH tissues, IL-7 and IL-15 genes are expressed to a significantly higher extent in localized PCA.

**Figure 2:**
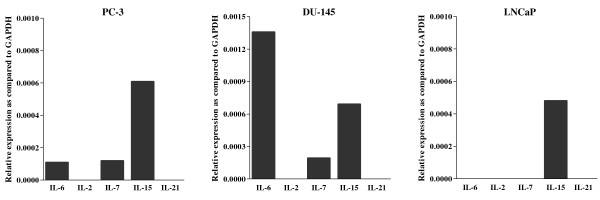
**Expression of IL genes in PCA cell lines **Total RNA was extracted from three PCA cell lines: PC-3 DU-145 and LNCaP. Following DNase treatment, RNA were reverse transcribed and resulting cDNA were analysed by quantitative real-time PCR for IL-2, IL-7, IL-15, IL-6 and IL-21 specific gene expression. Data are expressed as ratio to GAPDH gene expression.

IL-6 gene was previously found to be expressed by prostate cancer cells[44]. To address the issue of the nature of the cell types possibly expressing other cytokine genes in PCA tissues, we evaluated IL-2, IL-7, IL-15 and IL-21 gene expression in PC3, DU145 and LNCaP established PCA cell lines. Interestingly, we found that all cell lines expressed IL-15 gene, whereas IL-6 and IL-7 genes were only expressed in PC3 and DU145 cells (figure 2). In contrast, IL-2 and IL-21 genes were not expressed by any of the cell lines under investigation.

### Detection of c-γ cytokines in sera from patients with early stage PCA

High IL-6 serum titres have been observed in advanced PCA [42]. Furthermore, increased IL-7 serum titres have been reported in PCA[37], as compared to non age matched healthy donors, but no comparison with sera from patients with BPH was attempted, nor were patients with early stage PCA specifically studied. In order to verify, at the systemic level our findings from BPH and localized PCA tissues, we comparatively evaluated circulating titres of IL-6, IL-7 and IL-15 in pre-operative sera from patients with prostatic diseases.

**Figure 3:**
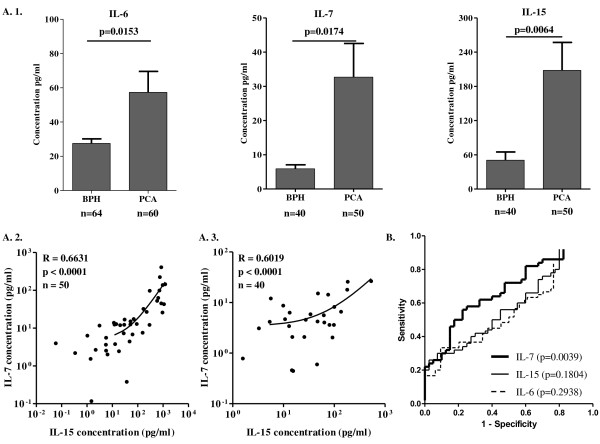
**Circulating levels of soluble factors in serum from patients bearing early stage PCA or BPH A**. Sera from patients bearing BPH or PCA were collected prior to surgery and tested for their content in the indicated factors by ELISA (A.1.). Statistical significance of differential titers in sera from patients bearing either BPH or PCA is also reported. Correlations between IL-7 and IL-15 levels in sera from patients with early stage PCA (A.2.) or BPH (A.3.) are displayed. **B**. ROC curves were created for all IL under investigation and their ability to distinguish between sera of patients with PCA or BPH was analyzed.

IL-6 was highly significantly increased even in sera from patients with pT1-2c stage PCA, as compared to sera from patients with BPH (average IL-6 concentration ± SEM = 57.34 ± 12.12 vs. 27.46 ± 2.62 pg/ml, p = 0.0153, figure 3A.1.). Most interestingly, serum levels of IL-7 and IL-15 were also significantly higher in patients with localized PCA as compared to patients with BPH (average IL-7 concentration ± SEM = 32.69 ± 9.73 vs. 5.87 ± 1.17 pg/ml, p = 0.0174 and average IL-15 concentration ± SEM 208.07 ± 48.50 vs. 50.51 ± 14.34 pg/ml, p = 0.0064, figure 3A.1.). Remarkably, a highly significant correlation was observed between IL-7 and IL-15 levels in sera from patients with either early stage PCA (p < 0.0001, R = 0.6631, n = 50) or BPH (p < 0.0001, R = 0.6019, n = 40) (Figure 3, panels A.2. and A.3.).

Notably, cytokine serum titres did not significantly correlate with the specific expression of the corresponding gene in autologous prostate tissues.

### IL-7 serum titres distinguish patients with BPH or PCA

ROC curves were constructed to verify whether serum IL titres could help distinguishing patients with localized PCA from patients with BPH (Figure 3B). Interestingly, although specific serum titres were significantly higher in patients with PCA, as compared with patients with BPH, areas under curve (AUC) were small for IL-6 (AUC: 0.5547, 95%CI: 0.4515-0.6579) and IL-15 (AUC: 0.5825, 95%CI: 0.4647-0.7003). Therefore, these cytokine titres were not helpful in identifying patients with early stage malignancies (p = 0.2938 and p = 0.1804, respectively). In contrast, analysis of the ROC curve for IL-7 (AUC: 0.6775, 95%CI: 0.5676-0.7874 p = 0.0039) indicated that specific IL titres were effectively distinguishing patients with early stage PCA from patients with BPH.

### Detection of acute phase proteins in sera from patients with early stage PCA

Serum biomarkers of systemic inflammation C reactive protein (CRP) and serum amyloid A (SAA), have been found to be associated with PCA [45-47]. Furthermore, HMGB1 gene has been suggested to be overexpressed in prostate PCA [48]. The corresponding gene product is a prominent member of a group of mediators released by damaged cells, collectively known as damage associated molecular patterns (DAMP)[49]. These factors might be responsible for the activation of the innate immune system, potentially resulting in cytokine gene expression and secretion[50].

We did not observe significant differences between sera from patients with early stage PCA and those from patients with BPH regarding CRP, SAA or HMGB1 levels (Table 1: average CRP concentration ± SEM 7.47 ± 1.92 μg/ml vs. 5.70 ± 1.16 μg/ml, p = 0.4370; average SAA concentration ± SEM 2.81 ± 0.31 vs. 2.71 ± 0.29 μg/ml, p = 0.7728; average HMGB1 concentration ± SEM 52.77 ± 12.48 vs. 49.11 ± 15.13 ng/ml, p = 0.7497). Notably, no significant correlation could be observed between IL-6, IL-7 or IL-15 serum levels and acute phase protein titres. Similarly, no correlation with PSA levels or Gleason score was detectable.

**Table 1:** Acute phase protein concentrations in sera from patients with early stage PCA and BPH

		CRP (μg/ml)	SAA (μg/ml)	HMGB1 (ng/ml)
**Early stage PCA**	**Average**	7.47	2.81	52.77
	**SEM**	1.92	0.31	12.48
	**n**	66	18	18

**BPH**	**Average**	5.70	2.71	49.11
	**SEM**	1.16	0.29	15.13
	**n**	66	19	19

**p**		0.4370	0.7728	0.7497

## Discussion

A large body of literature based on histopathological, epidemiological and molecular pathology data suggests that chronic inflammation might play an important role in prostate oncogenesis [2]. IL represent essential mediators of inflammation and titres of a number of them have been shown to be increased in patients bearing PCA[7]. Most of these studies, however, have addressed IL detection in patients with advanced stage cancers. In these conditions, high tumor burdens and mechanisms inherent with metastatic spread, e,g, osteolysis, might be involved in the induction of IL production. Furthermore, sterile inflammation primed by intratumoral ischemia and associated necrosis could also promote IL release [51]. Therefore, data from patients with advanced tumors are unlikely to provide useful information on the role eventually played by inflammation or immunosuppression in prostate oncogenesis. In order to gain insights into events occurring in early stages of prostate cancerogenesis, in this study we evaluated IL gene expression and protein production at local and systemic levels, respectively, in localized PCA, as compared with BPH.

Our data clearly indicate that the expression of a number of genes encoding pro-inflammatory and homeostatic IL, including IL-6, IL-7 and IL-15 is detectable more frequently and to a higher extent, in early stage PCA as compared to BPH tissues. Notably, the genes encoding these IL were also found to be expressed to different levels by established PCA cell lines.

To investigate the possibly systemic nature of the pro-inflammatory state detectable in PCA[37,52], we measured IL titres in sera from patients with BPH or early stage PCA. We report here for the first time that IL-7 and IL-15 serum levels are significantly higher in patients with localized PCA than in patients with BPH. IL-6 titres were also significantly higher in patients with PCA than in patients with BPH despite the early stage of their disease [53].

IL-6 is known to be produced by multiple cell types, including activated macrophages, smooth muscle cells and PCA cells [53]. Instead, IL-7 is produced by multiple stromal cell types and epithelial cells from different districts including thymus and gut[10]. Most recently IL-7 production has been detected in normal, but not in transformed prostate epithelial cells[36]. IL-15 is typically produced by activated antigen presenting cells of the myeloid lineage, including monocytes, macrophages and dendritic cells. However, IL-15 gene has also been found to be expressed by a multiplicity of other cell types, including stromal and epithelial cells[9]. Importantly, epithelial BPH cells have been shown to express IL-15[34]. In our patients, IL serum titres were not significantly correlated with specific gene expression in the autologous prostate tissues, thereby suggesting that cells other than tumor cells might be at least in part responsible for IL production. Thus, while we here show that IL-6, IL-7 and IL-15 genes are expressed in established PCA cell lines, future studies are warranted to clarify the cellular sources of these IL within the prostate and systemically.

The nature of the mechanisms promoting IL-6, IL-7 and IL-15 gene expression and protein production in PCA is also unclear. Different microorganisms, including virus and bacteria[54,55] have been suspected to contribute to oncogenesis in the prostate. Indeed, their presence could result in chronic inflammation. Alternatively, it has been suggested that direct damage of epithelial cells might be caused by toxic compounds contained in urines or by dietary factors[2]. Cell death might, in turn, result in the activation of the innate immune system by DAMPs [49]. Soluble factors produced by DAMP-triggered innate immune system cells, including IL-6, have repeatedly been suggested to promote prostate cancerogenesis[2]. However, here we show that at least circulating levels of HMGB1, SAA and CRP acute phase proteins are similar in patients with BPH or localized, early stage PCA.

Analysis of ROC curves indicates that IL-7, but neither IL-6 nor IL-15 titres do distinguish sera from patients with PCA from sera from patients with BPH. These findings might set the stage for larger studies also addressing the clinical relevance of IL-7 serum titres in the monitoring of PCA response to treatment and recurrences.

On the other hand, our data raise the issue of potential functional consequences of c- γ cytokine gene expression and secretion in PCA. Preliminary experiments indicate that none of the IL under investigation enhances the proliferation of cells from established PCA cell lines (data not shown). However, studies performed in breast cancer[56] indicate that IL-7 promotes the production of pro-angiogenic factors by tumor cells and proliferation of endothelial cells. IL-15 has also been shown to stimulate angiogenesis "in vivo"[57]. Furthermore, although IL-7 and IL-15 are known to promote survival and functional maturation of T cells, they have also been demonstrated to induce the expression of PD-1 "exhaustion" marker in these cells[58]. Indeed, PCA tissues have been shown to be infiltrated by T cells largely expressing PD-1, and, less frequently, PD-L1 and PD-L2[59,60]. These data are consistent with previous reports on the phenotypic characteristics of circulating and tissue infiltrating lymphocytes in chronic viral infections[61-64]. In this context, it is remarkable that we found that the expression of the IL-21 gene, that is of the gene encoding the cytokine preventing PD-1 mediated T cell exhaustion[21-23] is rarely detectable in PCA.

## Conclusions

Our work indicates that even in early stages of PCA development, genes encoding IL of pro-inflammatory significance are over-expressed in malignant tissues, as compared to BPH specimens, Importantly, corresponding proteins are detectable to higher levels in sera from patients with localized PCA as compared to patients with BPH, and, in particular, IL-7 titres might represent an additional marker of potential clinical relevance.

## List of abbreviations

AUC: area under curve; BPH: benign prostatic hyperplasia; CI: confidence interval; CRP: C-reactive protein; c-γ: common receptor γ chain; DAMP: damage associated molecular patterns; EERP: endoscopic extraperitoneal radical prostatectomy; ELISA: enzyme-linked immunosorbent assay; GAPDH: glyceraldehyde-3-phosphate dehydrogenase; HMGB1: high mobility group B1; IL: interleukin; mAb: monoclonal antibody; PCA: prostate cancer; PD-1: programmed death receptor-1; PD-L1 and -L2: programmed death ligand-1 and ligand-2; PSA: prostate specific antigen; qRT-PCR: quantitative real-time polymerase chain reaction; ROC: receiver operating characteristic; SAA: serum amyloid A; TUR-P: transurethral resection.

## Competing interests

The authors declare that they have no competing interests.

## Authors' contributions

All authors read and approved the final manuscript. CM, CLM, ET and KY participated in the design, acquisition and analysis of data. GCS and MP and SW conceived the study; GCS, MH and AB provided funding support and revised the manuscript critically for important intellectual content. LB made substantial contributions to the analysis and interpretation of data.
